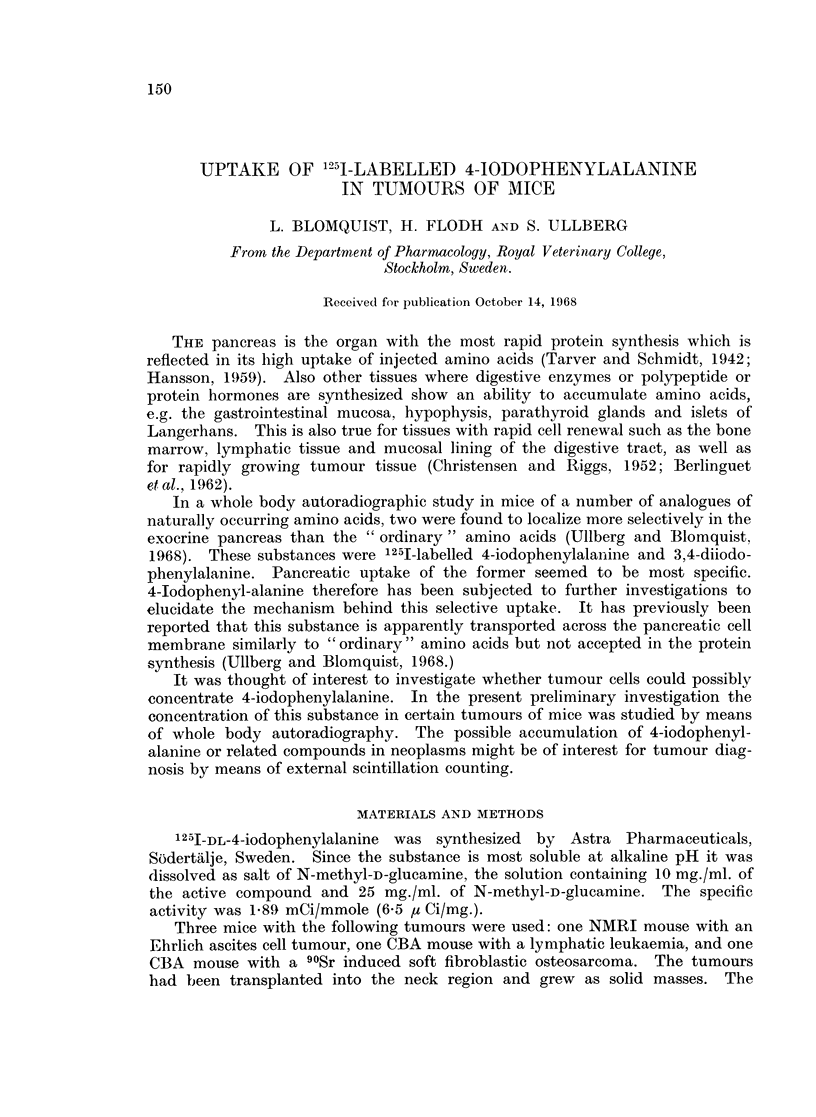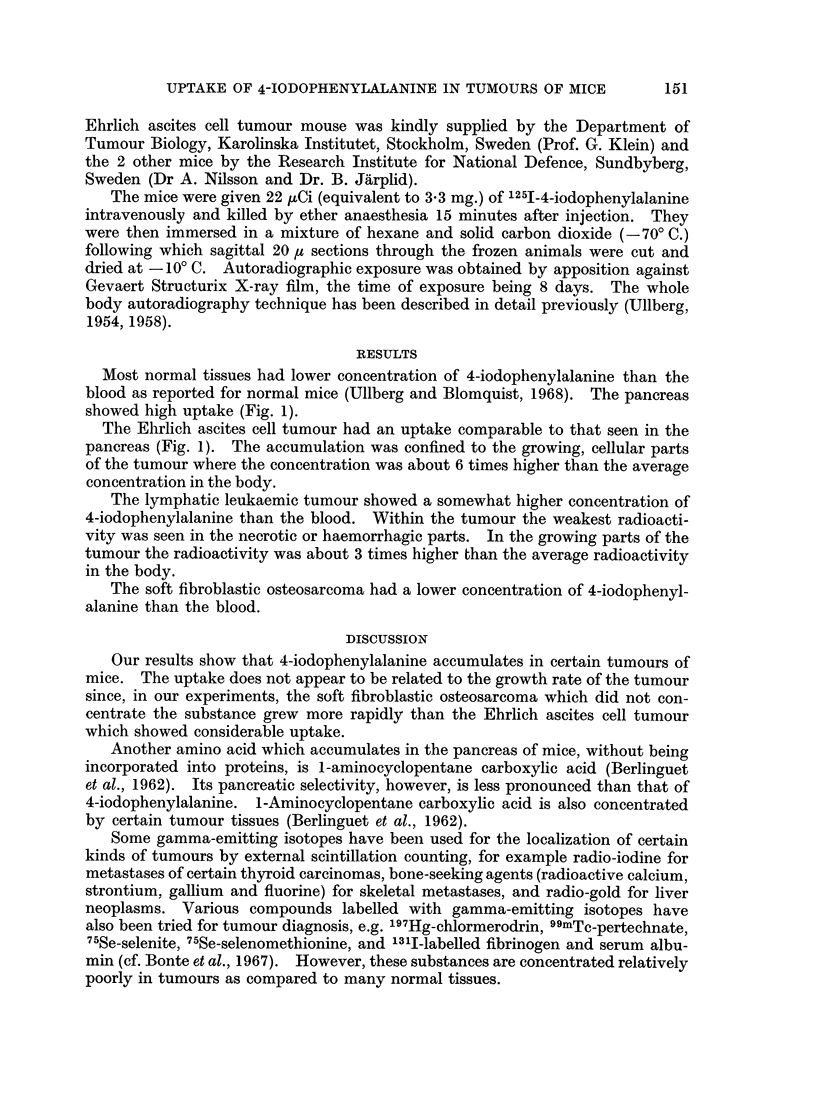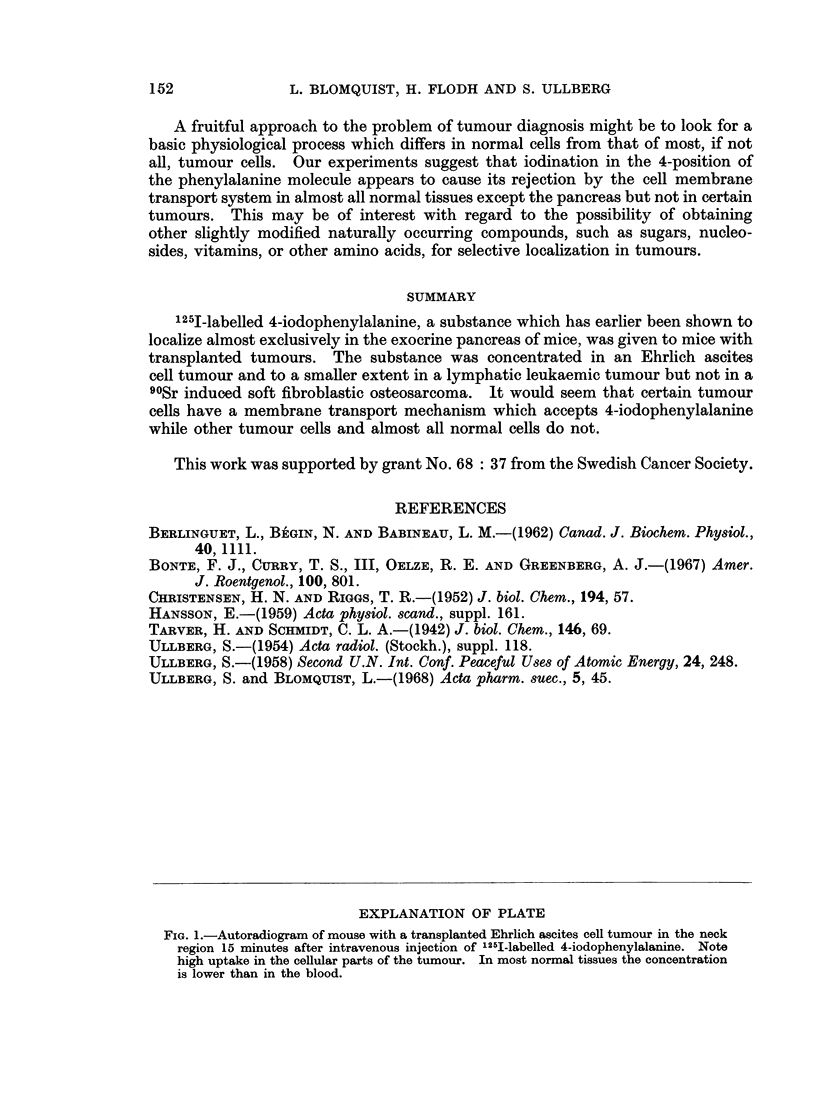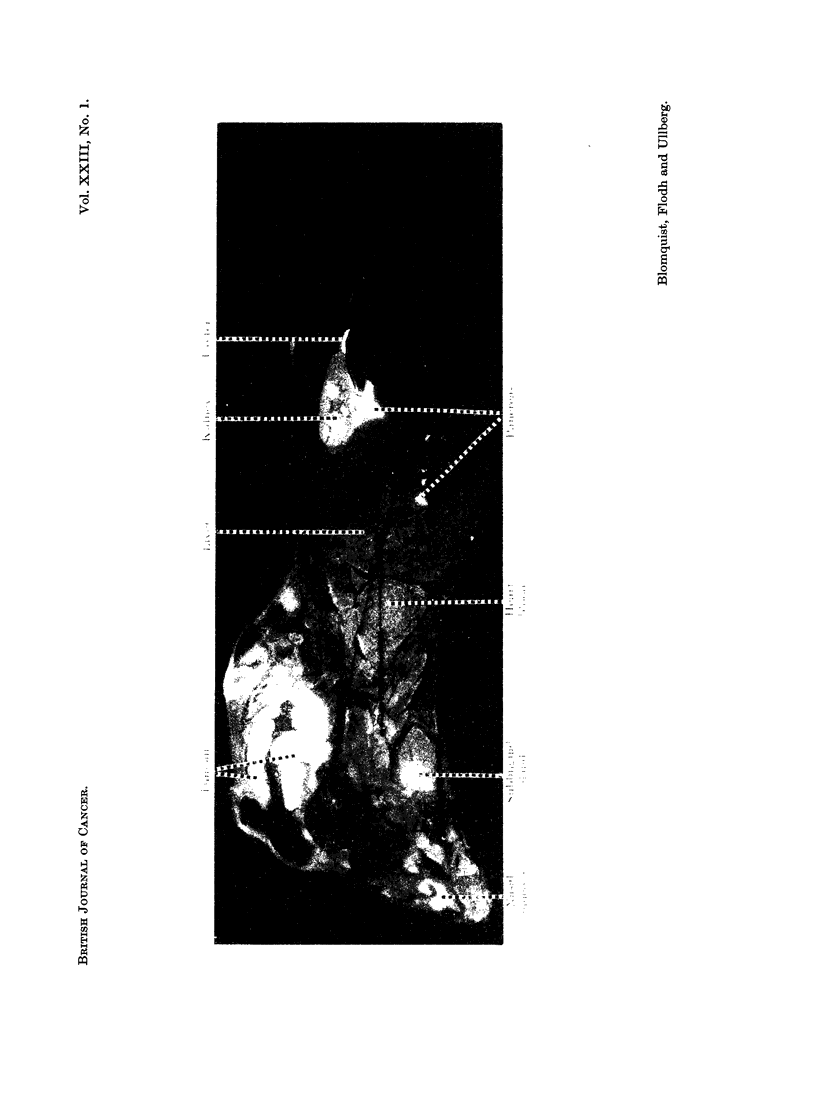# Uptake of 125I-labelled 4-iodophenylalanine in tumours of mice.

**DOI:** 10.1038/bjc.1969.22

**Published:** 1969-03

**Authors:** L. Blomquist, H. Flodh, S. Ullberg

## Abstract

**Images:**


### UPTAKE OF $^{125}$I-LABELLED 4-IODOPHENYLALANINE IN TUMOURS OF MICE

L. BLOMQUIST, H. FLODH AND S. ULLBERG

*From the Department of Pharmacology, Royal Veterinary College, Stockholm, Sweden.*

Received for publication October 14, 1968

THE pancreas is the organ with the most rapid protein synthesis which is reflected in its high uptake of injected amino acids (Tarver and Schmidt, 1942; Hansson, 1959). Also other tissues where digestive enzymes or polypeptide or protein hormones are synthesized show an ability to accumulate amino acids, e.g. the gastrointestinal mucosa, hypophysis, parathyroid glands and islets of Langerhans. This is also true for tissues with rapid cell renewal such as the bone marrow, lymphatic tissue and mucosal lining of the digestive tract, as well as for rapidly growing tumour tissue (Christensen and Riggs, 1952; Berlinguet *et al.*, 1962).

In a whole body autoradiographic study in mice of a number of analogues of naturally occurring amino acids, two were found to localize more selectively in the exocrine pancreas than the " ordinary " amino acids (Ullberg and Blomquist, 1968). These substances were $^{125}$I-labelled 4-iodophenylalanine and 3,4-diiodophenylalanine. Pancreatic uptake of the former seemed to be most specific. 4-Iodophenyl-alanine therefore has been subjected to further investigations to elucidate the mechanism behind this selective uptake. It has previously been reported that this substance is apparently transported across the pancreatic cell membrane similarly to "ordinary" amino acids but not accepted in the protein synthesis (Ullberg and Blomquist, 1968.)

It was thought of interest to investigate whether tumour cells could possibly concentrate 4-iodophenylalanine. In the present preliminary investigation the concentration of this substance in certain tumours of mice was studied by means of whole body autoradiography. The possible accumulation of 4-iodophenylalanine or related compounds in neoplasms might be of interest for tumour diagnosis by means of external scintillation counting.

### MATERIALS AND METHODS

$^{125}$I-DL-4-iodophenylalanine was synthesized by Astra Pharmaceuticals, Södertälje, Sweden. Since the substance is most soluble at alkaline pH it was dissolved as salt of N-methyl-D-glucamine, the solution containing 10 mg./ml. of the active compound and 25 mg./ml. of N-methyl-D-glucamine. The specific activity was 1·89 mCi/mmole (6·5 $\mu$ Ci/mg.).

Three mice with the following tumours were used: one NMRI mouse with an Ehrlich ascites cell tumour, one CBA mouse with a lymphatic leukaemia, and one CBA mouse with a $^{90}$Sr induced soft fibroblastic osteosarcoma. The tumours had been transplanted into the neck region and grew as solid masses. The

Ehrlich ascites cell tumour mouse was kindly supplied by the Department of Tumour Biology, Karolinska Institutet, Stockholm, Sweden (Prof. G. Klein) and the 2 other mice by the Research Institute for National Defence, Sundbyberg, Sweden (Dr A. Nilsson and Dr. B. Järplid).

The mice were given 22 $\mu$Ci (equivalent to 3·3 mg.) of $^{125}$I-4-iodophenylalanine intravenously and killed by ether anaesthesia 15 minutes after injection. They were then immersed in a mixture of hexane and solid carbon dioxide ($-70°$ C.) following which sagittal 20 $\mu$ sections through the frozen animals were cut and dried at $-10°$ C. Autoradiographic exposure was obtained by apposition against Gevaert Structurix X-ray film, the time of exposure being 8 days. The whole body autoradiography technique has been described in detail previously (Ullberg, 1954, 1958).

### RESULTS

Most normal tissues had lower concentration of 4-iodophenylalanine than the blood as reported for normal mice (Ullberg and Blomquist, 1968). The pancreas showed high uptake (Fig. 1).

The Ehrlich ascites cell tumour had an uptake comparable to that seen in the pancreas (Fig. 1). The accumulation was confined to the growing, cellular parts of the tumour where the concentration was about 6 times higher than the average concentration in the body.

The lymphatic leukaemic tumour showed a somewhat higher concentration of 4-iodophenylalanine than the blood. Within the tumour the weakest radioactivity was seen in the necrotic or haemorrhagic parts. In the growing parts of the tumour the radioactivity was about 3 times higher than the average radioactivity in the body.

The soft fibroblastic osteosarcoma had a lower concentration of 4-iodophenylalanine than the blood.

### DISCUSSION

Our results show that 4-iodophenylalanine accumulates in certain tumours of mice. The uptake does not appear to be related to the growth rate of the tumour since, in our experiments, the soft fibroblastic osteosarcoma which did not concentrate the substance grew more rapidly than the Ehrlich ascites cell tumour which showed considerable uptake.

Another amino acid which accumulates in the pancreas of mice, without being incorporated into proteins, is 1-aminocyclopentane carboxylic acid (Berlinguet *et al.*, 1962). Its pancreatic selectivity, however, is less pronounced than that of 4-iodophenylalanine. 1-Aminocyclopentane carboxylic acid is also concentrated by certain tumour tissues (Berlinguet *et al.*, 1962).

Some gamma-emitting isotopes have been used for the localization of certain kinds of tumours by external scintillation counting, for example radio-iodine for metastases of certain thyroid carcinomas, bone-seeking agents (radioactive calcium, strontium, gallium and fluorine) for skeletal metastases, and radio-gold for liver neoplasms. Various compounds labelled with gamma-emitting isotopes have also been tried for tumour diagnosis, e.g. $^{197}$Hg-chlormerodrin, $^{99m}$Tc-pertechnate, $^{75}$Se-selenite, $^{75}$Se-selenomethionine, and $^{131}$I-labelled fibrinogen and serum albumin (cf. Bonte *et al.*, 1967). However, these substances are concentrated relatively poorly in tumours as compared to many normal tissues.

A fruitful approach to the problem of tumour diagnosis might be to look for a basic physiological process which differs in normal cells from that of most, if not all, tumour cells. Our experiments suggest that iodination in the 4-position of the phenylalanine molecule appears to cause its rejection by the cell membrane transport system in almost all normal tissues except the pancreas but not in certain tumours. This may be of interest with regard to the possibility of obtaining other slightly modified naturally occurring compounds, such as sugars, nucleosides, vitamins, or other amino acids, for selective localization in tumours.

SUMMARY

$^{125}$I-labelled 4-iodophenylalanine, a substance which has earlier been shown to localize almost exclusively in the exocrine pancreas of mice, was given to mice with transplanted tumours. The substance was concentrated in an Ehrlich ascites cell tumour and to a smaller extent in a lymphatic leukaemic tumour but not in a $^{90}$Sr induced soft fibroblastic osteosarcoma. It would seem that certain tumour cells have a membrane transport mechanism which accepts 4-iodophenylalanine while other tumour cells and almost all normal cells do not.

This work was supported by grant No. 68 : 37 from the Swedish Cancer Society.

### REFERENCES

BERLINGUET, L., BÉGIN, N. AND BABINEAU, L. M.—(1962) *Canad. J. Biochem. Physiol.*, **40**, 1111.
BONTE, F. J., CURRY, T. S., III, OELZE, R. E. AND GREENBERG, A. J.—(1967) *Amer. J. Roentgenol.*, **100**, 801.
CHRISTENSEN, H. N. AND RIGGS, T. R.—(1952) *J. biol. Chem.*, **194**, 57.
HANSSON, E.—(1959) *Acta physiol. scand.*, suppl. 161.
TARVER, H. AND SCHMIDT, C. L. A.—(1942) *J. biol. Chem.*, **146**, 69.
ULLBERG, S.—(1954) *Acta radiol.* (Stockh.), suppl. 118.
ULLBERG, S.—(1958) *Second U.N. Int. Conf. Peaceful Uses of Atomic Energy*, **24**, 248.
ULLBERG, S. and BLOMQUIST, L.—(1968) *Acta pharm. suec.*, **5**, 45.

---

EXPLANATION OF PLATE

FIG. 1.—Autoradiogram of mouse with a transplanted Ehrlich ascites cell tumour in the neck region 15 minutes after intravenous injection of $^{125}$I-labelled 4-iodophenylalanine. Note high uptake in the cellular parts of the tumour. In most normal tissues the concentration is lower than in the blood.

Blomquist, Flodh and Ullberg.